# Childcare in Infancy and Later Obesity: a Narrative Review of Longitudinal Studies

**DOI:** 10.1007/s40124-017-0134-7

**Published:** 2017-06-15

**Authors:** Silvia Costa, Jean Adams, Sarah Gonzalez-Nahm, Sara E Benjamin Neelon

**Affiliations:** 1 UKCRC Centre for Diet and Activity Research (CEDAR), MRC Epidemiology Unit, School of Clinical Medicine, University of Cambridge, Cambridge, UK; 2 Department of Health, Behavior and Society, Johns Hopkins Bloomberg School of Public Health, Johns Hopkins University, Baltimore, MD USA

**Keywords:** Child care, Nurseries, Infant, Adiposity, Body weight

## Abstract

**Purpose of Review:**

The purpose of this review was to summarize the current literature on the longitudinal relationship between non-parental childcare during infancy and later obesity.

**Recent Findings:**

Eleven studies met the inclusion criteria, comprising 74 associations relevant to the review. Studies were highly heterogeneous in terms of defining childcare, categorizing different types of childcare, assessing obesity, and age at measurement of outcome and exposure. Most of the associations were either non-significant (42 associations, 57%) or showed a significant association between increased exposure to childcare and greater obesity (30 associations, 41%). There were very few examples of associations indicating that childcare was associated with lower obesity.

**Summary:**

There is limited research on the longitudinal relationship between childcare in infancy and later obesity. Existing studies showed mixed results, similar to recent reviews reporting on cross-sectional studies and older ages. The different definitions of childcare and wide variety of measures of exposure make comparisons between studies challenging.

## Introduction

High rates of childhood obesity are a worldwide concern [1]. Infancy has been repeatedly highlighted as a critical or sensitive period in post-natal life for the development and prevention of obesity [2–4]. Obesity and rapid weight gain during infancy are significantly associated with increased risk of obesity, type II diabetes, and hypertension during both childhood and adulthood [2, 4, 5]. Several individual (e.g., sex, and diet behavior), inter-personal (e.g., feeding practices), and environmental factors (e.g., attending childcare) have been reported to influence the early development of childhood obesity [6]. As environmental factors affect large numbers of children, they represent potential targets for obesity prevention at the population level [7].

A rising number of children attend out-of-home childcare starting as early as the first year of life [8], with many spending much of their week days in these settings [8, 9]. Around one quarter of children up to 3 years old in developed countries attend some form of childcare [10]. Among offspring of employed mothers in the USA, an estimated 15.9% of infants aged less than 1 year and 29.8% aged one to 2 years attended an organized childcare facility (e.g., day care center or nursery) in 2011, and roughly 35% of infants received care from a grandparent [8]. The same report estimated that children aged ≤4 years with employed mothers spent an average of 36 h per week in childcare, varying from 23 h with grandparents to 33 h in childcare centers [8]. In contrast, few infants were estimated to receive non-parental care in their own homes—4% of those aged <1 year and 6% of those aged 1–2 years [8]. As such, childcare settings have become targets for intervention efforts to prevent obesity in early life [11, 12].

A number of studies have examined the relationship between childcare attendance and later obesity and found mixed results [13•, 14•]. The association may depend, in part, on the age when children first enter childcare, their total exposure, and the timing of measurement of both exposure and outcomes [13•]. Because infancy is a period of rapid physical development [2] and vulnerability [4], where children are dependent on others [15], it is crucially important to investigate childcare settings as a potential risk factor for the development of obesity. In this narrative review, we sought to examine and summarize the current evidence on the longitudinal relationship between childcare in infancy and adiposity, body mass, and obesity.

## Methods

We performed a narrative review, with clear a priori definitions of participants, exposures, and outcomes of interest.

### Eligibility Criteria

#### Participants

We included studies with children aged birth to 24 months at the time of measurement of exposure to childcare, as this is a commonly accepted age range for the infancy period [2]. This is also an increasingly recognized critical period for the development of obesity and its health consequences both in infancy and in later life [16, 17].

We included studies based in middle- and high-income countries; we excluded studies in low-income countries, using the World Bank’s definition of a gross national income per capita of less than US$1025 [18]. We excluded studies from these countries because of the differing economic, social and health environments in higher- versus lower-income settings, including investment in and access to health and education [19, 20] and leading causes of death [21]. These factors are likely to influence both the quality and use of childcare.

### Study Design

We included only observational longitudinal studies, including case-control, prospective and retrospective cohort studies, and excluded all other study designs. We focused on observational designs to allow the exploration of associations between childcare and the outcomes of interest as they exist in the general population and in real-life settings, rather than in experimental situations and settings. Including only longitudinal studies helped reduce the possibility of reverse causality between receipt of childcare and obesity.

### Exposure and Comparator

The exposure of interest was non-parental childcare, versus a comparator of parental care. If the exposure was measured as a categorical variable (e.g., parental care and formal non-parental childcare), the comparator of interest was parental care. In some cases authors used the term “no childcare” and we assumed this to be parental care. If the exposure was measured as a continuous variable (e.g., number of hours of non-parental childcare per week), we extracted change in outcome per unit increase in change in exposure. Where possible, we did not impose any standard definitions of specific types of childcare and instead used the authors’ language. In one case, after discussion with the author, we grouped all care provided in someone else’s or the child’s own home as “informal care” irrespective of who provided it [9].

### Outcomes

The outcomes of interest included adiposity or body mass (“obesity”) directly measured by researchers, childcare staff or parents, or overweight or obesity, at a time point subsequent to recording of childcare use. Examples of accepted measures included weight, body mass index (BMI), waist or hip circumference, weight-for-length (WFL) ratio, skinfold thickness, fat mass, and overweight or obesity status. We excluded studies using self-reported measures.

### Analyses

We only included studies that used multivariable analysis to account for potential confounders, such as maternal smoking during pregnancy, birth weight, duration of breastfeeding, and socioeconomic status. This decision was based on existing evidence that these variables are associated with obesity in both childhood and adulthood [4, 5] and may be associated with the decision to use non-parental childcare and so have the potential to confound any relationship between childcare and obesity.

### Literature Search and Synthesis

One author (SC) conducted the literature search using a three-stage process. Firstly, she scanned the bibliography of recent literature reviews [13•, 14•] covering the relationship between childcare in preschool age children and overweight and obesity. From these, she identified potentially relevant studies for which full texts were retrieved and screened against the eligibility criteria. Next, she scanned the bibliographies of included studies, identified potentially relevant studies, and retrieved and screened full texts against the eligibility criteria. There were no searches of publication databases. We included both papers published in peer-reviewed journals and published theses. Theses are generally considered by two examiners and revised accordingly—a process arguably similar to peer review of journal articles. Finally, we searched the Google Scholar for citations of included studies identified in the first two steps. We then identified potentially relevant studies and retrieved and screened full texts against the eligibility criteria. We then tabulated key study information and performed a narrative synthesis of results, exploring patterns according to type of childcare, timing of measurement of childcare exposure, timing of measurement of obesity-related outcomes, and country of origin.

## Results

### Summary of Included Studies

We included 11 studies—two thesis [22, 23] and nine peer-reviewed articles [9, 24–31]—that met the eligibility criteria. All studies were published within the last 10 years (2008–2016) and were based in high-income countries [18], with over one third of the studies (*n* = 4) originating from the USA (Table 1). Sample sizes varied widely (range 105 to 27,821 participants), with no studies restricted by child sex, race or ethnicity. All studies reported on more than one relevant association, resulting from using more than one measure of either exposure, outcome or both; measuring exposure, outcome or both at more than one time point; and sub-group analyses. Therefore, although only 11 studies met the inclusion criteria, we included 74 relevant associations. As exposure and outcome measures were very heterogeneous between and within studies, with sometimes multiple exposure and outcome measures in the same study, we report our results at the level of individual associations rather than at the study-level for the remainder of the review. We believe that this helps both to demonstrate the heterogeneity in the literature, detail the full extent of knowledge in this area, and make progress towards exploring sources of heterogeneity in reported results.

**Table 1:** Characteristics of studies included in the review

Study (date)	Location	Sample size	Sex	Race/ethnicity/country of birth	Socioeconomic indicator
Benjamin Neelon et al. (2015)	Denmark	27,821	41% females	Predominantly white and born in Denmark	Mean household income: 51–53,000 Danish Kroner.
Benjamin et al. [9]	USA	1138 (933 for 3y outcome)	50% females	15% Black, 67% White, 5% Hispanic, 13% other	Annual household income: 13% < US$40000, 23% US$40000–70,000, 64% > US$70000
Gubbels et al. [25]	Netherlands	2396	49% female	97% born in Netherlands	Maternal education level: 62% above median, 38% below median
Isong et al. [26]	USA	4700 (2400 for OLS sensitivity analysis)	48.3% females	16% African-American, 55% White 23% Hispanic, 3% Asian, 3% other	Annual household income: 34% < US$25000, 29% US$25000–50,000; 37% > US$50000
Kim and Peterson [27]	USA	8150 overall (~285^a^ pre-term and LBW infants ~7140^a^ term and NBW infants)	Not reported	Not reported	Not reported
Lehto et al. [28]	Finland	1683	Not reported	Not reported	Not reported
Lin et al. [29]	Hong Kong	3584	51% female	100% Chinese (63% of mothers born in Hong Kong)	Highest parental education: 28% < grade 9; 43% grade 10–11; 29% > grade 11
Mathai [22]	USA	105	67% female	29% Black, 44% White, 18% Mixed, 8% Asian, 8% Hispanic, 2% American Indian/Alaska Native	Mean household income: US$13390
Metzer [23]	Canada	3525	49% female	16% not born in Canada, 84% born in Canada	Maternal education: 12% less than high school, 14% high school, 27% some post high school, 47% college/university graduate
Pearce et al. (2010)	UK	12,354	Not reported	87% white British, 2% other white, 1% mixed, 2% Indian, 4% Pakistani, 3% Black or Black British, 2% other	Maternal educational qualifications: 15% none, 56% school level, 29% post-school level
Scully et al. [31]	Ireland	273	52% female	Not reported	Maternal education: 60% university level

*LBW* low birth weight, *NBW* normal birth weight, *OLS* ordinary least-squares, *y* years

^a^Approximate numbers calculated from the sub-group sample percentages and the total sample size presented in paper, where the latter was rounded up to the nearest 50

The most common exposure variable was duration of childcare exposure (*n* = 26 associations, 35%) [9, 24, 27, 31], measured as either time per week or years, or categorized as full- versus part-time attendance. The next most frequent was a binary measure of any childcare use or not (*n* = 21 associations, 28%) [22, 25, 26, 29], where parental care was the main comparator. Age at childcare exposure was 0–12 months for 82% of the associations studied [9, 22, 24, 25, 27–30]. The most common outcomes were weight gain (*n* = 24 associations, 32%—although weight gain was only included as an outcome in one study) [27]; overweight or obese status (*n* = 19 associations, 26%) [23–25, 28–30]; and BMI *z* score (*n* = 19 associations, 26%) [9, 24–26, 29]. None of the studies included crossing weight percentiles as outcomes. Both overweight or obese status and BMI *z* score were calculated using a variety of different growth references and standards (Table 2). Outcome variables were measured at 0–12 months in 42 (57%) of the associations studied [9, 22, 24, 25, 27], with 11 years [29] being the oldest age at outcome measurement.

**Table 2:** Analyses and results of studies included in the review

Study (date)	Exposure	Age at exposure	Outcome	Age at outcome	Adjustments in most adjusted model	Results of most adjusted model
Benjamin Neelon et al. (2015)	Attend any child care (increment of days/month)	0–12 m	BMI *z* score (WHO growth standards)	12 m	Maternal age, smoking during pregnancy, parity, maternal pre-pregnancy BMI, household income, breastfeeding, infant birth weight	*β* = 0.03 (95%CI 0.01 to 0.05); *p* = 0.003
	Attend any child care (increment of days/month)	0–12 m	Overweight (WHO growth standards)	12 m	As above	OR = 1.05 (95%CI: 1.01 to 1.10); *p* = 0.047
	Attend daycare (increment of days/month)	0–12 m	BMI *z* score (WHO growth standards)	12 m	As above	*β* = 0.03 (95%CI: 0.01 to 0.05); *p* = 0.02
	Attend daycare (increment of days/month)	0–12 m	Overweight (WHO growth standards)	12 m	As above	OR = 1.05 (95%CI: 0.99 to 1.10); *p* = 0.06
	Attend crèche (increment of days/month)	0–12 m	BMI *z* score (WHO growth standards)	12 m	As above	*β* = 0.05 (95%CI: 0.01 to 0.10); *p* = 0.03
	Attend crèche (increment of days/month)	0–12 m	Overweight (WHO growth standards)	12 m	As above	OR = 1.05 (95%CI: 0.95 to 1.16); *p* = 0.35
	Attend age-integrated care (increment of days/month)	0–12 m	BMI *z* score (WHO growth standards)	12 m	As above	*β* = 0.08 (95%CI: 0.04 to 0.12); *p* < 0.001
	Attend age-integrated care (increment of days/month)	0–12 m	Overweight (WHO growth standards)	12 m	As above	OR = 1.15 (95%CI: 1.06 to 1.27); *p* = 0.001
Benjamin et al. [9]	Attend any childcare (increment of 10 h/week)	0–6 m	Weight for length *z* score (CDC growth reference)	1 y	Child’s age, gender, race/ethnicity; maternal pre-pregnancy BMI, smoking during pregnancy, parity; paternal BMI; household income. Additional potential mediators: breastfeeding duration; child’s sleep duration, television viewing	*β* = 0.08 (95%CI: 0.01 to 0.15); *p* = 0.03 ᅟ ᅟ Additional mediators adjusted: *β* = 0.07 (95%CI: −0.001 to 0.15); *p* = 0.05
	Attend any childcare (increment of 10 h/week)	0–6 m	BMI *z* score (CDC growth reference)	3 y	As above	*β* = 0.09 (95%CI: 0.02 to 0.16); *p* = 0.01 Additional mediators adjusted: *β* = 0.06 (95%CI: −0.02 to 0.15); *p* = 0.16
	Attend childcare center (increment of 10 h/week)	0–6 m	Weight for length *z* score (CDC growth reference)	1 y	Child’s age, gender, race/ethnicity; maternal pre-pregnancy BMI, smoking during pregnancy, parity; paternal BMI; household income	*β* = 0.03 (95%CI: 0.08 to 0.14); *p* = 0.62
	Attend childcare center (increment of 10 h/week)	0–6 m	BMI *z* score (CDC growth reference)	3 y	As above	*β* = 0.04 (95%CI: 0.08 to 0.16); *p* = 0.51
	Attend childcare in someone else’s home (increment of 10 h/week)	0–6 m	Weight for length *z* score (CDC growth reference)	1 y	As above	*β* = 0.11 (95%CI: 0.01 to 0.20); *p* = 0.02
	Attend childcare in someone else’s home (increment of 10 h/week)	0–6 m	BMI *z* score (CDC growth reference)	3 y	As above	*β* = 0.12 (95%CI: 0.02 to 0.21); *p* = 0.02
	Attend childcare in child’s own home (increment of 10 h/week)	0–6 m	Weight for length *z* score (CDC growth reference)	1 y	As above	*β* = 0.02 (95%CI: 0.09 to 0.13); *p* = 0.73
	Attend childcare in child’s own home (increment of 10 h/week)	0–6 m	BMI *z* score (CDC growth reference)	3 y	As above	*β* = 0.03 (95%CI: 0.08 to 0.15); *p* = 0.56
Gubbels et al. [25]	Attend any childcare vs no childcare	7 m	BMI *z* score (Dutch national reference)	1 y	Maternal hours of employment, overweight, educational level, age country of birth; parental hours of employment, overweight, alternative lifestyle; child’s birth weight; breastfeeding duration (backward deleted in order of significance)	*β* = 0.02; *p* > 0.05
	Attend any childcare vs no childcare	7 m	BMI *z* score (Dutch national reference)	2 y	As above	*β* = 0.08; *p* > 0.05
	Attend any childcare vs no childcare	7 m	Change in BMI *z* score (Dutch national reference)	1–2 y	As above	*β* = 0.02; *p* > 0.05
	Attend any childcare vs no childcare	7 m	Overweight (≥85th percentile Dutch national reference)	1 y	As above	OR = 1.32 (95%CI: 1.04 to 1.69); *p* < 0.05
	Attend any childcare vs no childcare	7 m	Overweight (≥85th percentile Dutch national reference)	2 y	As above	OR = 1.18 (95%CI: 0.89 to 1.57); *p* > 0.05
	Attend any childcare vs no childcare	7 m	Change from normal weight to overweight vs remaining normal weight	1–2 y	As above	OR = 1.36 (95%CI: 0.95 to 1.94); *p* > 0.05
	Attend any childcare vs no childcare	1 y	BMI *z* score (Dutch national reference)	2 y	As above	*β* = 0.12; *p* < 0.05
	Attend any childcare vs no childcare	1 y	Change in BMI *z* score (Dutch national reference)	1–2 y	As above	*β* = 0.10; *p* < 0.05
	Attend any childcare vs no childcare	1 y	Overweight (≥85th percentile Dutch national reference)	2 y	As above	OR = 1.17 (95%CI: 0.88 to 1.55); *p* > 0.05
	Attend any childcare vs no childcare	1 y	Change from normal weight to overweight vs remaining normal weight	1–2 y	As above	OR = 1.37 (95%CI: 0.96 to 1.95); *p* > 0.05
Isong et al. [26]	Attend any non-parental childcare vs parental care	24 m	BMI *z* score (CDC growth reference)	Kindergarten entry	Child age, race/ethnicity, sex, maternal age, maternal weight (in kg), household structure, family SES, and neighborhood safety [OLS]	*β* = 0.08 (SE = 0.03); *p* = 0.01
	Attend any non-parental childcare vs parental care	24 m	BMI *z* score (CDC growth reference)	Kindergarten entry	As above [OLS sensitivity changes in exposure across waves]	*β* = 0.05 (SE = 0.06); *p* = 0.39
	Attend any non-parental childcare vs parental care	24 m	BMI *z* score (CDC growth reference)	Kindergarten entry	As above [OLS sensitivity lagged effects]	*β* = 0.01 (SE = 0.02); *p* = 0.66
	Attend any non-parental childcare vs parental care	24 m	BMI *z* score (CDC growth reference)	Kindergarten entry	As above plus time varying covariates: employment status, change in residence, household structure, and SES for each wave of data. [individual fixed effects model]	*β* = 0.02 (SE = 0.02); *p* = 0.41
	Attend any non-parental childcare vs parental care	24 m	BMI *z* score (CDC growth reference)	Kindergarten entry	Child age, race/ethnicity, sex, maternal age, maternal weight (in kg), household structure, family SES, and neighborhood safety. [instrumental variable estimation]	*β* = 1.12 (SE = 0.76); *p* = 0.14
Kim and Peterson [27]	Age at initiation of childcare vs parental care (all infants)	0–2.9 m	Weight gain	9 m	Child’s age, sex, race/ethnicity, birth weight, prematurity; household poverty; maternal education level, employment, smoking, pre-pregnancy BMI; parental marital status; breastfeeding initiation; early introduction of solid foods	*β* = 136 g (95%CI: 40 to 232); *p* > 0.05
	Age at initiation of childcare vs parental care (all infants)	3–5.9 m	Weight gain	9 m	As above	*β* = 155 g (95%CI 87 to 225); *p* < 0.05
	Age at initiation of childcare vs parental care (all infants)	>6 m	Weight gain	9 m	As above	*β* = 93 g (95%CI: 30 to 156); *p* > 0.05
	Attend childcare center vs parental care (all infants)	0–9 m	Weight gain	9 m	As above	*β* = 79 g (95%CI: 14 to 143); *p* > 0.05
	Attend non-relative childcare vs parental care (all infants)	0–9 m	Weight gain	9 m	As above	*β* = 204 g (95%CI: 82 to 327); *p* > 0.05
	Attend relative childcare vs parental care (all infants)	0–9 m	Weight gain	9 m	As above	*β* = 162 g (95%CI:106 to 219); *p* > 0.05
	Attend any childcare <35 h/week vs none (all infants)	0–9 m	Weight gain	9 m	As above	*β* = 175 g (95%CI: 100 to 250); *p* > 0.05
	Attend any childcare >35 h/week vs none (all infants)	0–9 m	Weight gain	9 m	As above	*β* = 139 g (95%CI: 83 to 195); *p* > 0.05
	Age at initiation of childcare vs parental care (pre-term and low birth weight infants)	0–2.9 m	Weight gain	9 m	Child’s age, sex, race/ethnicity; household poverty; maternal education level, employment, smoking, pre-pregnancy BMI; parental marital status; breastfeeding initiation; early introduction of solid foods	*β* = 204 g (95%CI: 112 to 296); *p* < 0.05
	Age at initiation of childcare vs parental care (pre-term and low birth weight infants)	3–5.9 m	Weight gain	9 m	As above	*β* = 204 g (95%CI: 15 to 393); *p* > 0.05
	Age at initiation of childcare vs parental care (pre-term and low birth weight infants)	>6 m	Weight gain	9 m	As above	*β* = 259 g (95%CI: 131 to 388); *p* > 0.05
	Attend childcare center vs parental care (pre-term and low birth weight infants)	0–9 m	Weight gain	9 m	As above	*β* = 369 g (95%CI: 296 to 442); *p* > 0.05
	Attend non-relative childcare vs parental care (pre-term and low birth weight infants)	0–9 m	Weight gain	9 m	As above	*β* = 92 g (95%CI: −243 to 426); *p* > 0.05
	Attend relative childcare vs parental care (pre-term and low birth weight infants)	0–9 m	Weight gain	9 m	As above	*β* = 174 g (95%CI: 79 to 269); *p* > 0.05
	Attend any childcare <35 h/week vs none (pre-term and low birth weight infants)	0–9 m	Weight gain	9 m	As above	*β* = 109 g (95%CI: −106 to 325); *p* > 0.05
	Attend any childcare >35 h/week vs none (pre-term and low birth weight infants)	0–9 m	Weight gain	9 m	As above	*β* = 270 g (95%CI: 176 to 365); *p* > 0.05
	Age at initiation of childcare vs parental care (term and normal birth weight infants)	0–2.9 m	Weight gain	9 m	As above	*β* = 163 g (95%CI: 105 to 222); *p* < 0.05
	Age at initiation of childcare vs parental care (term and normal birth weight infants)	3–5.9 m	Weight gain	9 m	As above	*β* = 147 g (95%CI: 123,171); *p* < 0.01
	Age at initiation of childcare vs parental care (term and normal birth weight infants)	>6 m	Weight gain	9 m	As above	*β* = 112 g (95%CI: 89 to 136); *p* < 0.05
	Attend childcare center vs parental care (term and normal birth weight infants)	0–9 m	Weight gain	9 m	As above	*β* = 67 g (95%CI: 14 to 120); *p* > 0.05
	Attend non-relative childcare vs parental care (term and normal birth weight infants)	0–9 m	Weight gain	9 m	As above	*β* = 225 g (95%CI: 120 to 330); *p* < 0.05
	Attend relative childcare vs parental care (term and normal birth weight infants)	0–9 m	Weight gain	9 m	As above	*β* = 177 g (95%CI: 111 to 243); *p* < 0.05
	Attend any childcare <35 h/week vs none (term and normal birth weight infants)	0–9 m	Weight gain	9 m	As above	*β* = 192 g (95%CI: 111 to 243); *p* < 0.05
	Attend any childcare >35 h/week vs none (term and normal birth weight infants)	0–9 m	Weight gain	9 m	As above	*β* = 144 g (95%CI: 53 to 236); *p* > 0.05
Lehto et al. [28]	No exposure to childcare	not entered	Overweight (IOTF cut-offs)	3 and 5 y combined	Child’s age, gender, birth weight; maternal BMI, smoking during pregnancy; paternal BMI; highest education in the family, family structure	OR = 1.41 (95%CI: 0.77 to 2.58)
	Entered childcare at <1 y	<1 y	Overweight (IOTF cut-offs)	3 and 5 y combined	As above	OR = 3.13 (95%CI: 1.62 to 6.03); *p* = 0.003
	Entered childcare between 1–2 y	1–1.99 y	Overweight (IOTF cut-offs)	3 and 5 y combined	As above	OR = 1.37 (95%CI: 0.70 to 2.66)
Lin et al. [29]	Attend informal childcare vs parental care	6 m	Change in BMI *z* score (WHO growth standards)	11 y	Child’s sex and birth weight *z* score, maternal birthplace (Hong Kong born or not), highest parental education and occupation, monthly household income per head, and the interaction of maternal birthplace and highest parental education	*β* = 0.07 (95%CI: –0.02 to 0.17)
	Attend informal childcare vs parental care	6 m	Overweight (IOTF cut-offs)	11 y	As above	OR = 1.13 (95%CI: 0.97 to 1.33)
Mathai [22]	Attend any childcare vs parental care	0–8 m	Weight for length *z* score (WHO growth standards)	1 y	Maternal pre-pregnancy BMI, maternal age, maternal education, child baseline and follow-up age, child birth weight, child birth order, household income, household size, infant formula, and breastfeeding duration; maternal single status, maternal CC use (parental care: <10 h/week non-parental care; center care: >10 h/week non-parental care), child gender and race/ethnicity, and age at solid introduction	Infants in parental care had higher weight for length *z* score mean = 1.83 vs. −0.05; *p* = 0.05
	Attend any childcare vs parental care	0–8 m	Weight for age *z* score (WHO growth standards)	1 y	As above	Infants in parental care had higher weight for age *z* score mean = 1.20 vs. −0.34; *p* = 0.01
	Attend any childcare vs parental care	0–8 m	Change in weight for length *z* score (WHO growth standards)	1 y	As above	Infants in parental care had a greater change in WFL *z* score *β* = 2.06 (SE = 1.04); *p* = 0.05
	Attend any childcare vs parental care	0–8 m	Change in weight for age *z* score (WHO growth standards)	1 y	As above	Infants in parental care had a greater change in WFA *z* score *β* = 1.69 (SE = 0.66); *p* = 0.01
Metzer [23]	Attend informal childcare vs parental care	0–2 y	Overweight (IOTF cut-offs)	8–10 y	Maternal age at birth, pre-pregnancy BMI, smoking during pregnancy, marital status and education, full-time employment during infancy and toddlerhood, number of siblings in the household, immigrant status, infant size for gestational age, and breastfeeding	OR = 0.93 (95%CI: 0.62 to 1.41)
	Attend formal childcare vs parental care	0–2 y	Overweight (IOTF cut-offs)	8–10 y	As above	OR = 0.93 (95%CI: 0.67 to 1.31)
Pearce et al. (2010)	Attend informal childcare vs parental care	0–4 m	Overweight (IOTF cut-offs)	3 y	Maternal age at first live birth, ethnicity, number of children in household at age 9 months, maternal pre-pregnancy overweight, birth weight, maternal smoking during pregnancy, National Statistics Socio-Economic Classification, lone parenthood, maternal education, breastfeeding duration, timing of introduction of solid foods.	OR = 1.05 (95%CI: 0.90 to 1.24)
	Attend formal childcare vs parental care	0–4 m	Overweight (IOTF cut-offs)	3 y	As above	OR = 1.02 (95%CI: 0.79 to 1.33)
	Attend informal childcare vs parental care	0–36 m	Overweight (IOTF cut-offs)	3 y	As above	OR = 1.18 (95%CI: 0.99 to 1.41)
	Attend formal childcare vs parental care	0–36 m	Overweight (IOTF cut-offs)	3 y	As above	OR = 1.07 (95%CI: 0.82 to 1.41)
Scully et al. [31]	Attend any childcare (unit of exposure not specified)	0–2 y	Abdominal circumference	2 y	Infant birth weight, gender, study group, duration of breastfeeding, age at follow-up; maternal BMI at follow-up, education	*β* = 0.215 (95%CI: 0.005 to 0.038); *p* = 0.010
	Attend any childcare (unit of exposure not specified)	0–2 y	Thigh skinfold	2 y	As above	*β* = 0.194 (95%CI: 0.003 to 0.050); *p* = 0.025
	Attend any childcare (unit of exposure not specified)	0–2 y	Sum of all skinfolds	2 y	As above	*β* = 0.203 (95%CI: 0.007 to 0.077); *p* = 0.019
	Attend any childcare (unit of exposure not specified)	0–2 y	Waist-to-height ratio	2 y	As above	*β* = 0.178 (95%CI: 0.000 to 0.000); *p* = 0.036

*BMI* body mass index, *CDC* Centers for Disease Control, *CI* confidence interval; *IOTF* International Obesity Task Force, *m* months, *OLS* ordinary least-squares, *OR* odds ratio, *OW* overweight, *SE* standard error, *WHO* World Health Organization, *y* years

Potential confounders included in multivariable analyses were relatively consistent among studies, and included indicators of socioeconomic status (e.g., household income and parental education level), maternal characteristics (e.g., age, pre-pregnancy BMI, and smoking during pregnancy), and child’s characteristics and early life factors (e.g., gender, birth weight, and breastfeeding duration). However, only four studies [24, 26, 28, 30] explicitly attempted to investigate the potential mediating role of some of these factors, such as breastfeeding duration. Results were highly heterogeneous. For example, some studies reported positive associations between attending childcare and risk of later overweight or obesity [24, 25] or increased adiposity indicators [24–27], while others reported negative [22] or no association [23, 27, 30].

### Synthesis of Findings

#### Exposure to Any Versus No Childcare

Seven studies included analyses of any non-parental childcare (undifferentiated further by authors) versus parental childcare [9, 22, 24–26, 29, 31]. Out of 29 relevant associations 14 (48%) were not significant, 11 (38%) showed significant positive associations, and four (14%) showed significant negative associations. Considering the method of exposure measurement (e.g., continuous versus binary), or outcome measurement (e.g., overweight status or BMI *z* score) did not reveal any particular patterns.

#### Exposure to Specific Types of Childcare

Associations between exposure to informal childcare and adiposity outcomes were assessed in five studies [9, 23, 24, 27, 30]. Out of 15 associations, five (33%) were significant and positive. The remaining ten (67%) were not significant. No discernible patterns were seen in relation to type of outcome measured. Five studies included assessment of the association between exposure to formal childcare and outcomes of interest. Of 12 relevant associations, the majority (*n* = 9, 75%) were non-significant, and only three (25%) showed a positive and significant relationship. Perhaps of note, all of these significant associations were reported in the same study [24].

#### Age at Measurement of Childcare Exposure

Exposure to childcare was measured at <12 months in the majority of associations (*n* = 57, 77%). Of these 35 (61%) were not significant, 18 (32%) associations were significant and positive, and four (7%) showed a significant and negative effect. Of the 17 associations that included a measurement of childcare exposure at ≥12 months, 12 (70%) were not significant, with the remaining five (30%) showing significant positive associations.

#### Age at Measurement of Outcome

Age at measurement of outcome was 0–24 months in most studies (*n* = 54 associations, 73%). Of these, 28 (52%) associations were not significant, 22 (41%) were significant and positive, and the remaining four (7%) were significant and negative. In the 16 (22%) associations where age of measurement was 25–71 months, 11 (69%) were not significant and five (31%) were significant positive associations. No significant associations were observed between childcare and overweight or adiposity indicators measured at ≥72 months (6 years), although such long-term follow-up was only included in four (5%) associations from two studies [23, 29].

#### Country of Origin

In studies originating from the USA (four studies; 41 associations) [9, 22, 26, 27], most associations were non-significant (*n* = 26, 63%). However, in 11 cases (27%) there was a significant positive association, and the remaining four (11%) reported a significant negative association. In studies from countries other than the USA (seven studies; 33 associations) [23–25, 28–31], 18 (55%) associations were non-significant, and 15 (45%) were significant and positive.

## Discussion

We used reproducible methods to conduct a narrative review of the longitudinal association between childcare exposure during infancy and later adiposity. We found 11 studies that met our inclusion criteria, reporting a total of 74 relevant associations. Overall, the majority of associations were non-significant or showed positive associations indicating that increased exposure to childcare was associated with greater obesity. There were very few examples of significant negative associations, where increased childcare exposure was associated with lower obesity or adiposity indicators, and these were all from the same study [22]. The authors propose that the food provided in childcare (more processed foods) compared to parental care (more in line with infant feeding guidelines) may, paradoxically, explain this difference. However, it is also possible that these “inconsistent” findings merely reflect expected variation. Studies were highly heterogeneous in terms of defining childcare, categorizing different types of childcare, assessing obesity and adiposity, and age at measurement of outcome and exposure. Although we attempted to group studies and associations meaningfully (e.g., by type of exposure), this did not reveal any clear patterns.

One striking finding from the review is the heterogeneity of the exposures studied. While some authors compared any non-parental care to parental care, others focus on particular types of care, seeking to differentiate between, for example, informal and formal care, and different sub-categories within these. Furthermore, the definitions of different types of childcare were not consistent. For example, different authors use “informal childcare” to mean both unlicensed but paid-for care, grandparental care, and some center-based care. These differences in how the exposure was defined undoubtedly contributed to some of the heterogeneity in findings. Development of a clear typology of childcare and definitions of different types of care, alongside clear reporting of what types of care are considered in different studies would likely add some clarity to the current literature. However, international differences in types of childcare available may complicate this process.

The heterogeneity of findings in this review suggests that any effect of childcare in infancy on the development of obesity may not be large, although even a small effect at the individual level may have significant implications at the population level. Specific factors related to the quality and context of the childcare environment may be important. The nutritional and physical activity environments provided by different types of childcare, and by different providers within broad types, can vary greatly [32–34]. Although all included studies controlled for a variety of known confounders, none considered the potential mediating or moderating roles of childcare-specific factors such as dietary provisions (other than breastfeeding), sleep, or physical activity opportunities. Thus, the variability in childcare environments may partly explain the heterogeneity of study findings.

It is also possible that the effects of childcare on obesity vary according to child and family characteristics, such as socioeconomic position. There is expanding evidence that some public health interventions are more effective in affluent groups [35] and that some risk factors are more potent in less affluent groups [36]. Again, such effects may obscure findings at the wider group level, such as those studied here. Further consideration of child- and family-level variables as effect modifiers, rather than just confounders may yield valuable insights.

In addition to variations in effect according to family characteristics, use of some types of childcare may be restricted to particular types of families. In these cases, lack of variability in family characteristics within childcare types may mean that statistical adjustment for family characteristics is inadequate and that uncontrolled confounding by family characteristics persists. For example, in five studies looking at the use of formal childcare, only one [24] found positive associations with overweight or BMI *z* score. The latter was set in Denmark, where paid parental leave can be taken for up to 52 weeks and shared by both parents. Danish parents using crèches or age-integrated facilities in the first year of their child’s life may, therefore, be a select group with particular characteristics (e.g., ineligible for paid parental leave because they are recent immigrants). These characteristics may themselves influence the development of obesity and so act as confounders in any relationship between childcare and obesity [37]. Thus, the reported significant positive associations between formal childcare and both overweight and BMI *z* score may be a reflection of uncontrolled confounding, rather than a reflection of the influence of this type of childcare in the development of obesity.

We did not find strong evidence that exposure to childcare in the first year of life was any more or less harmful than exposure in the second year. However, given the variety of ways in which exposure was assessed, and the number of different outcome measures used, it is difficult to draw clear conclusions. It is possible that younger children are no more vulnerable to the effects of childcare than older children. However, we did not find any studies that tested this hypothesis directly and further work is required to assess whether any effects of childcare on obesity vary according to the timing of exposure.

We did find some indication that the effects of childcare in infancy on obesity may diminish over time. While only four associations in included studies included follow-up of children beyond 6 years, none of these were statistically significant. Thus, it may be possible that any harmful effects of childcare in infancy are not sustained into later childhood or beyond. Further longitudinal studies, with follow-up into middle childhood and beyond, are required to assess any effects of early childcare on later obesity.

One potential mechanism linking childcare and obesity is the early discontinuation of breastfeeding. Exclusive breastfeeding and breastfeeding duration of a minimum of four to 6 months have been consistently linked to a lower likelihood of obesity in childhood [38–40]. Research has shown that women who return to work within the first 4 months postpartum and need to rely on out-of-home childcare are less likely to continue breastfeeding [41–44]. Two papers included in this review found that breastfeeding did not mediate the association between childcare and obesity [23, 30]; however, neither of these examined exclusive breastfeeding. The timing of mothers’ return to work is influenced, in part, by the amount of maternity leave available to mothers—which varies globally. While adequate maternity leave policies may help encourage women to breastfeed their infants, they are only one of multiple factors affecting a woman’s ability and decision to exclusively breastfeed. Childcare policies that support continued breastfeeding [45] may help women continue to exclusively breastfeed their infants in childcare, which may result in lower rates of child obesity over time.

### Limitations

While we used reproducible methods to find relevant papers for inclusion in this review, we did not conduct a systematic review. In particular, we did not conduct an exhaustive search of relevant databases such as MEDLINE, CINAHL or Embase. We may not, therefore, have included all relevant studies meeting the eligibility criteria. Furthermore, by including published theses, we may have incorporated some less rigorously assessed material than may be found in peer-reviewed journal articles. However, these theses make an important contribution to what is currently a very small volume of relevant literature in this area. In addition, we did not perform all aspects of searching and screening in duplicate which may have led to additional errors. We are currently conducting a systematic review examining the association of childcare factors with diet, physical activity, sedentary behavior, sleep, and stress that will help overcome these limitations [46]. We also restricted the review to longitudinal data in order to reduce the possibility of reverse causality [47]. However, longitudinal studies alone cannot determine causality [48] and we did not conduct a formal quality assessment of included studies. While it would likely be very difficult to gain consent and parental agreement to conduct a randomized controlled trial of, e.g., parental versus non-parental care, a variety of more innovative methods could be used to study the effects of childcare on obesity—including quasi-experimental evaluations of changes in national and local childcare and parental leave policies. The studies included in our review were also all conducted in high-income countries, and one third were from the USA alone. Thus, our findings may not be generalizable to other countries not represented, particularly middle-income countries.

## Conclusions

Although our results were heterogeneous, they suggest the association between childcare and obesity is likely to be neutral or harmful in terms of later adiposity and obesity. As such, there is a clear need for further research on this topic. Longitudinal research is resource intensive and researchers must often rely on existing cohort studies, with questions and variables, which were not guided by any specific research questions at the outset. While we recognize this limitation on what can be studied, it does not diminish the need to ensure that future studies are informed by theory. Further studies should also seek to include longer follow-up periods to determine whether, how, for whom, and under what circumstances any harmful effects of childcare on obesity persist into later childhood and beyond.

Although we cannot conclude from the available evidence that childcare is definitely a risk factor for obesity, we found almost no evidence that it is protective. Until it is clarified whether childcare has a neutral or harmful effect on obesity, it is important to ensure that all childcare settings promote the health of the children in childcare, particularly in terms of the quality and quantity of foods offered and opportunities for physical activity.

Overall, we found few longitudinal studies exploring the association between childcare in infancy with later obesity. Further research is required to develop a typology of childcare and stronger conceptual theory about how childcare may influence the development of obesity. These theories could be used to drive future testing of pre-specified hypotheses concerning when, how, for whom and under what circumstances childcare in infancy may lead to obesity in later life. Studies with longer follow-up into later childhood and beyond are also required to determine any long-term public health impact of childcare. The available evidence is neither comprehensive nor consistent enough to warrant concern about infants using childcare. However, given the increasing numbers of children attending childcare and the amount of time children spend in these settings, it is sensible to ensure childcare environments promote the health of all children.
